# Preventive application of *Delftia acidovorans* suppresses *Pseudomonas tolaasii*-induced brown blotch in *Pleurotus ostreatus*

**DOI:** 10.3389/fmicb.2026.1881575

**Published:** 2026-06-30

**Authors:** Ying Luo, Wenting Shi, Hanbing Liu, Wenjie Jia, Nuerziya Yalimaimaiti, Qi Zhu, Chenyi Lin, Peisong Jia

**Affiliations:** 1 College of Horticulture, Xinjiang Agricultural University, Urumqi, China; 2 Key Laboratory of Integrated Pest Management on Crops in Northwestern Oasis, Xinjiang Key Laboratory of Agricultural Biosafety, Ministry of Agriculture and Rural Affairs, National Plant Protection Scientific Observation and Experiment Station of Korla, Institute of Plant Protection, Xinjiang Uygur Autonomous Region Academy of Agricultural Sciences, Urumqi, China

**Keywords:** antagonistic bacteria, biological control, delftibactin, secondary metabolite biosynthesis, siderophore, whole-genome sequencing

## Abstract

Brown blotch disease of *Pleurotus ostreatus,* caused by *Pseudomonas tolaasii,* substantially restricts mushroom production. Current control methods have drawbacks, including chemical residues and pathogen resistance. To address this issue, we isolated a bacterium from surface-sterilized, mildly diseased fruiting bodies, designated strain 24b, and evaluated its biocontrol potential against *P. tolaasii*. *In vitro* dual culture assays showed that strain 24b produced a stable inhibition zone against *P. tolaasii*. In bag cultivation experiments, preventive application of strain 24b prior to pathogen inoculation significantly reduced disease severity, with control efficacy markedly higher than that of curative application. The strain was identified as *Delftia acidovorans* by 16S rRNA gene sequencing and whole-genome analysis. Genomic analysis revealed multiple secondary metabolite biosynthetic gene clusters, including a complete delftibactin A/B cluster associated with siderophore synthesis. The presence of these clusters may provide a genetic basis for the observed antagonistic activity. Safety assessments showed no hemolytic activity or genes encoding known virulence factors. These findings indicate that *D. acidovorans* strain 24b is a promising biocontrol agent against brown blotch disease, potentially acting through niche competition and antibiosis.

## 1 Introduction

*Pleurotus ostreatus,* commonly known as the oyster mushroom, is one of the most widely cultivated edible mushrooms worldwide, valued for its nutritional content, short growth cycle, and high biological efficiency (Sánchez, 2010). However, intensive cultivation practices have inadvertently created favorable conditions for pathogen outbreaks. Brown blotch disease, primarily caused by *Pseudomonas tolaasii*, was first described in *Agaricus bisporus* more than a century ago (Osdaghi et al., 2019). Subsequent research confirmed that *P. tolaasii* also infects *P. ostreatus* (Suyama and Fujii, 1993), where it causes significant yield losses and severe quality degradation, characterized by sunken, yellow-to-brown lesions that render fruiting bodies unmarketable (Azu Okorley et al., 2019; Zhang et al., 2013). Since its initial discovery, this disease has remained a major biological constraint for the oyster mushroom industry, highlighting the urgent need for effective and sustainable management strategies.

Current management relies heavily on chemical bactericides. However, the availability of effective and safe bactericides against *P. tolaasii* is limited, and concerns persist regarding chemical residues on harvested mushrooms and the potential development of pathogen resistance (Osdaghi et al., 2019; Yuan et al., 2024). Consequently, biological control agents (BCAs) have garnered increasing attention. Several antagonistic bacteria, such as *Bacillus* spp., *Pseudomonas* spp., and *Mycetocola* spp. have been isolated from mushroom-associated environments and shown to inhibit *P. tolaasii* under laboratory conditions (Aslani et al., 2018; Fermor and Lynch, 1988; Hermenau et al., 2020; Jafaripour et al., 2024; Lee et al., 2014; Tajalipour et al., 2014; Tsukamoto et al., 2014). Nevertheless, the field efficacy of these BCAs is often inconsistent, partly due to poor colonization of the mushroom surface and limited compatibility with the host microenvironment (Lee et al., 2014; Osdaghi et al., 2019). Moreover, most studies have focused on *A. bisporus*, with relatively few conducted on *P. ostreatus*.

Recent advances in host-microbiome interactions have demonstrated that plants and other eukaryotes can selectively promote beneficial microbes under pathogen attack, which help establish local defense barriers (Chen et al., 2020; Wang and Song, 2022). Applying this microecological framework to edible fungi, the epidermal tissues of mildly diseased *P. ostreatus* fruiting bodies may represent a unique ecological niche where the resident microbial community naturally restricts the progression of *P. tolaasii*. Therefore, isolating candidate strains directly from this endogenous defensive microbiota offers a promising alternative for obtaining indigenous, host-adapted antagonists, potentially circumventing the colonization failures commonly encountered with conventional BCAs.

One genus that remains largely unexplored in mushroom disease management is *Delftia*. *Delftia acidovorans* is a metabolically versatile bacterium commonly found in soil, water, and plant rhizospheres (Bhat et al., 2022). It has been studied for its ability to degrade organic pollutants (Khanthong et al., 2026; Shetty et al., 2015) and, more recently, for its plant growth-promoting and biocontrol activities against several soil-borne phytopathogens (Da Silveira et al., 2019; Islam et al., 2023; Perry et al., 2017; Han et al., 2017; Liu et al., 2022). These antagonistic and growth-promoting traits rely on several distinct mechanisms, including the production of diffusible and volatile antimicrobial metabolites (Da Silveira et al., 2019), the secretion of siderophores (Morel et al., 2016; Da Silveira et al., 2019), the degradation of quorum-sensing signals to attenuate pathogen virulence (Maisuria and Nerurkar, 2015), and the synthesis of phytohormones such as indole-3-acetic acid (Da Silveira et al., 2019). However, no study has examined the interaction between *Delftia* spp. and edible fungi, nor has any *Delftia* strain been tested for biocontrol of *P. tolaasii*.

Therefore, this study aimed to isolate indigenous antagonistic bacteria from mildly diseased *P. ostreatus* fruiting bodies. The biocontrol efficacy of the most promising isolate, *D. acidovorans* 24b, was evaluated under both *in vitro* and *in vivo* conditions. Additionally, the optimal timing for its application was determined, and the genomic basis of its antagonistic potential was investigated through whole-genome sequencing.

## 2 Materials and methods

### 2.1 Isolation of potential antagonistic bacteria from *Pleurotus ostreatus* fruiting bodies

Fruiting bodies of cultivated *Pleurotus ostreatus* exhibiting mild symptoms of brown blotch disease were collected in April 2024 from a mushroom cultivation facility in Urumqi, Xinjiang, China (43.46° N, 87.40° E). Tissue blocks (approximately 5 × 5 × 5 mm) excised from the margins of blotch lesions were surface-sterilized with 75% ethanol for 30 s, rinsed three times with sterile distilled water, and macerated in 1 mL of sterile water (Liu et al., 2022). The resulting suspension was serially diluted (10^−1^ to 10^−4^), and 100 μL aliquots from each dilution were spread onto Luria-Bertani (LB) agar and nutrient agar (NA) plates. The plates were incubated at 28 ± 1 °C for 3–5 days. Following incubation, single colonies with distinct morphologies were selected and subcultured on LB agar plates to obtain pure strains. Purified isolates were preserved in 20% (v/v) glycerol at −80 °C for long-term storage and on LB agar slants at 4 °C for short-term use.

### 2.2 *In vitro* screening for antagonistic strains against *Pseudomonas tolaasii*

#### 2.2.1 Bacterial strains and culture conditions

The target pathogen, *Pseudomonas tolaasii* strain Pt-36a, was originally isolated from diseased *P. ostreatus* fruiting bodies and preserved in the laboratory culture collection. For each assay, the pathogen was activated by streaking onto LB agar plates and incubating at 28 °C for 48 h. A single colony was then inoculated into LB broth and cultured overnight at 28 °C with shaking at 180 rpm. The bacterial suspension was adjusted to an optical density of 0.8 ± 0.01 at OD600, corresponding to approximately 10^8^ CFU/mL.

#### 2.2.2 Two-step screening for antagonistic strains

Antagonistic strains against *P. tolaasii* were screened using a two-step procedure (Fermor and Lynch, 1988; Geels and Schippers, 1983) with minor modifications.

##### 2.2.2.1 Primary screening using the inhibition zone method

A suspension of *P. tolaasii* (OD600 = 0.8) was evenly spread onto LB agar plates using a sterile cotton swab and allowed to dry under aseptic conditions for 15 min. Four candidate strains were then spot-inoculated onto each plate in a cross-shaped pattern using sterile pipette tips, with sterile water as a negative control. Each treatment was performed in triplicate. The plates were incubated at 25 °C for 48 to 72 h. Strains that formed distinct inhibition zones around the inoculated spots were selected as potential antagonists for secondary screening.

##### 2.2.2.2 Secondary screening by well diffusion assay

The potential antagonists were further evaluated using the agar well diffusion method. LB agar was autoclaved, cooled to approximately 45 °C, and mixed with a 1% (v/v) suspension of *P. tolaasii* (OD600 = 0.8). The inoculated medium was poured into sterile Petri dishes (20 mL per dish) and allowed to solidify. Wells (8 mm in diameter) were created in the center of each plate using sterile Oxford cups, and 100 μL of each potential antagonist culture (OD600 = 0.8) was added to the wells. A 6% kasugamycin (KSM) solution, diluted 1,000-fold, served as the chemical control, while sterile water was used as the negative control. Five independent plates were used per treatment (*n* = 5 plates). All plates were incubated at 25 °C for 72 h, after which the inhibition zone diameters (including the wells) were measured using a digital caliper.

### 2.3 *In vivo* biocontrol assay on cultivated *P. ostreatus*

#### 2.3.1 Substrate preparation and mushroom cultivation

The substrate for *P. ostreatus* cultivation consisted of cottonseed hulls (91%), wheat bran (5%), lime (2%), gypsum (1%), and calcium carbonate (1%), with a final moisture content of 65%. After thorough mixing, the substrate was packed into polypropylene cultivation bags and sterilized by autoclaving at 121 °C for 2 h. Bags measuring 12 cm × 24 cm were used for the initial screening experiment (Section 2.3.3), while larger bags (18 cm × 38 cm) were used for the application timing experiment (Section 2.3.4). All bags were fitted with a breathable paper patch for gas exchange. Once cooled, the bags were inoculated with *P. ostreatus* spawn and kept in darkness at 22 ± 1 °C for mycelial colonization. Colonized bags were then transferred to a fruiting chamber for fruiting body induction under controlled conditions: 10–22 °C, 85–90% relative humidity, and CO_2_ concentration below 600 ppm.

#### 2.3.2 Preparation of bacterial suspensions

Bacterial strains (*P. tolaasii* Pt-36a and four putative biocontrol strains) stored at −80 °C were streaked onto LB agar and incubated at 25 °C for 24 h. Single colonies were then inoculated into LB broth and cultured at 25 °C with shaking at 180 rpm for 12 h to obtain seed cultures. Subsequently, 100 μL of each seed culture was transferred into 100 mL of LB broth and incubated under the same conditions for 12 h. All bacterial suspensions were adjusted to an optical density of 0.8 ± 0.01 at 600 nm (OD600).

#### 2.3.3 Experimental treatments for curative and preventive application modes

When the diameter of *P. ostreatus* pilei reached 2.0–3.0 cm, the following treatments were applied: blank control (CK1), inoculation with sterile water only; negative control (CK2), inoculation with *P. tolaasii* suspension only; positive control (KSM), treated with 6% kasugamycin; treatment 1 (T1, curative mode), pathogen inoculation followed 24 h later by biocontrol strain suspension; and treatment 2 (T2, preventive mode), biocontrol strain suspension followed 24 h later by pathogen inoculation. For each treatment, 30 μL of bacterial suspension was evenly spread evenly onto the pileus surface using a sterile cotton swab. Each treatment was applied to 3–6 pilei per bag, with 10 replicate bags per treatment.

#### 2.3.4 Disease assessment and efficacy calculation

Disease severity on each pileus was evaluated 24 h after inoculation using a 0–5 scale based on symptom development (Supplementary Figure 1). The scale was defined as follows: 0 = no lesions; 1 = slight tissue discoloration or a water-soaked appearance, with no yellow lesions; 2 = obvious yellowing of the pileus, flat surface without depression; 3 = scattered punctate sunken lesions, lesions not coalescing; 4 = confluent sunken lesions affecting less than 50% of the area, with few or no bacterial exudates; 5 = large yellowish-brown sunken lesions affecting 50% or more of the area, with obvious bacterial exudate.

The Disease Severity Index (DSI) was calculated using the following formula:


$$\mathrm{DSI}=\frac{\sum (\;\text{Number of pilei}\;\mathrm{at}\;\text{each scale}\times \text{scale value})}{\text{Total pilei surveyed}\times \text{maximum scale value}}\times 100$$

For each cultivation bag, the DSI was calculated based on the 3–6 pilei within that bag, and this bag-level DSI was treated as a single biological replicate for statistical analysis.

The biocontrol efficacy was determined as follows:


$$\begin{array}{l} \text{Biocontrol efficacy}\;(\%)\\ =\frac{\mathrm{DSI}\;\text{of negative control}-\mathrm{DSI}\;\text{of treatment}\;}{\mathrm{DSI}\;\text{of negative control}\;}\times 100\% \end{array}$$

### 2.4 Evaluation of application timing on control efficacy of strain 24b

To determine the optimal application timing, strain 24b was applied at nine time points relative to *P. tolaasii* inoculation: 24, 18, 12, and 6 h before inoculation (pre-inoculation); simultaneously (0 h); and 6, 12, 18, and 24 h after inoculation (post-inoculation). Pathogen inoculation and strain 24b application followed the methods described in Sections 2.3.2 and 2.3.3. Each treatment was applied to 3–6 pilei per bag, with 3–6 replicate bags per treatment. Disease assessment and efficacy calculation were performed as described in Section 2.3.3. For each bag, a bag-level DSI was calculated based on the disease ratings of all pilei within that bag, and this bag-level DSI served as one biological replicate for statistical analysis.

### 2.5 Characterization of biocontrol, plant growth-promoting, and host-compatibility traits of strain 24b

#### 2.5.1 Assay of the biological control and mushroom growth-promoting traits of strain 24b

All biological control and mushroom growth-promoting (MGP) traits of strain 24b were qualitatively assessed using standard procedures and selective growth media. Siderophore production was detected on chrome azurol S (CAS) agar plates, where a color change from blue to orange indicated a positive result (Li et al., 2018; Schwyn and Neilands, 1987). Hemolytic activity was evaluated on blood agar plates containing 8% sheep blood (Schottmüller, 1903). Protease activity was determined on Yeast Extract Mannitol (YEM) agar supplemented with 5% (v/v) skim milk; a clear halo around the colony indicated a positive reaction (Brown and Foster, 1970). Cellulase activity was assessed on carboxymethyl cellulose (CMC) agar plates followed by Congo red staining, with the formation of a clear zone indicating CMC hydrolysis (Li et al., 2018; Teather and Wood, 1982). Glucanase activity was evaluated using *Poria cocos* medium (Zhang et al., 2023).

For MGP traits, nitrogen fixation ability was assessed using Ashby’s nitrogen-free medium (Wang et al., 2024). Indole-3-acetic acid (IAA) production was detected using the Salkowski colorimetric assay (Bric et al., 1991). The ability to produce 1-aminocyclopropane-1-carboxylate (ACC) deaminase was confirmed by growth on medium containing ACC as the sole nitrogen source, with no growth observed on nitrogen-free medium (Penrose and Glick, 2003). Phosphorus and potassium solubilization abilities were determined using National Botanical Research Institute’s Phosphate (NBRIP) and Alexandrov agar plates, respectively (Nautiyal, 1999; Rajawat et al., 2016). After incubation at 28 °C for 5 to 7 days, the plates were examined for the formation of clear zones around the bacterial colonies.

#### 2.5.2 Compatibility and safety assessment of strain 24b on P. Ostreatus

##### 2.5.2.1 Mycelial growth assay

The effect of the cell-free culture filtrate from strain 24b on the mycelial growth of *P. ostreatus* was evaluated on PDA plates. Strain 24b was cultured in LB broth at 28 °C for 72 h. The cell-free filtrate was prepared by centrifugation at 8,000 × g for 10 min at 4 °C, followed by filtration through a 0.22-μm membrane. The filtrate was then mixed with molten PDA to obtain final concentrations of 0, 1, 2.5, and 5% (v/v). A mycelial plug (5 mm in diameter), taken from the actively growing margin of a *P. ostreatus* colony, was placed at the center of each plate. Plates were incubated at 25 °C in the dark for 7 days. The mycelial growth front was marked on the bottom of each plate at 2 days post-inoculation, and a second mark was made on day 7. The distance between the two marks was measured, and the mycelial growth rate was calculated by dividing this distance by 5 days. Six independent plates were used per concentration.

##### 2.5.2.2 Fruiting body safety assay

*P. ostreatus* was cultivated as described in Section 2.3.1. Immature fruiting bodies with a pileus diameter of 2–3 cm were selected. Four treatments were applied by spreading the respective liquids onto the pilei, as detailed in Section 2.3.3: (i) sterile water (negative control, CK); (ii) *P. tolaasii* suspension at 10^8^ CFU/mL (positive control, Pt); (iii) sequential application of strain 24b suspension (10^8^ CFU/mL) followed by *P. tolaasii* suspension at the same concentration (preventive treatment, 24b + Pt); and (iv) strain 24b suspension at 10^8^ CFU/mL alone (safety treatment, 24b). Each treatment was applied to 3–5 pilei per bag, with 3–4 replicate bags per treatment.

Disease severity was scored, and the DSI was calculated as described in Section 2.3.3. For each bag, a bag-level DSI was calculated based on the disease ratings of all pilei within that bag, and this bag-level DSI served as one biological replicate for statistical analysis. Additionally, the pileus diameter of each individual fruiting body was recorded both at the time of treatment application and after 24 h. The growth increment was calculated by subtracting the initial from the final diameter. Subsequently, fruiting bodies were harvested and weighed individually to determine the final fresh weight per fruiting body. For growth increment and fresh weight, the values from all pilei within each bag were averaged to obtain bag-level means, and these bag-level means were used as biological replicates for statistical analysis (*n* = 3–4 bags per treatment).

### 2.6 Identification of strain 24b

#### 2.6.1 Morphological and physiological-biochemical characterization of strain 24b

The colony color and morphology of strain 24b were recorded after growth on LB agar at 28 °C for 48 h. Gram staining was performed according to Coico (2006). Scanning electron microscopy (SEM) images were obtained using a HITACHI SU8100 microscope (Hitachi High-Technologies Corp., Tokyo, Japan). Carbon source utilization, enzyme activities, and other conventional physiological and biochemical tests were performed according to the standard methods described in Bergey’s Manual of Systematic Bacteriology.

#### 2.6.2 Molecular identification of strain 24b

Genomic DNA of strain 24b was extracted using a Bacterial DNA Kit (Solarbio, China) following the manufacturer’s protocol. The quality and quantity of the extracted DNA were assessed using 1.0% (w/v) agarose gel electrophoresis and a NanoDrop 2000 spectrophotometer (Thermo Fisher Scientific, Waltham, MA, USA). The 16S rRNA gene was amplified from the genomic DNA by PCR using the universal primers 27F (5’-AGAGTTTGATCCTGGCTCAG-3′) and 1492R (5’-GGTTACCTTGTTACGACTT-3′). The amplified gene fragments were sequenced by Sangon Biotech Co., Ltd. (Shanghai, China). The obtained sequences were analyzed by comparison with reference sequences from the GenBank database using BLASTN. Phylogenetic analysis was performed using the Maximum Likelihood method under the K2 + G + I model in MEGA 7.0.

### 2.7 Whole-genome sequencing, assembly, annotation, and taxonomic identification

#### 2.7.1 DNA extraction and sequencing

A single colony of strain 24b was inoculated into LB broth and incubated at 28 °C for 12 h with shaking (Yoon et al., 2017). DNA was extracted using a standard SDS-phenol-chloroform method (Sambrook et al., 1989). DNA quality and quantity were assessed using a Qubit 3.0 fluorometer and a NanoDrop One spectrophotometer (Thermo Fisher Scientific, Waltham, MA, USA). Whole-genome sequencing was performed by Wuhan Bena Biotechnology Co., Ltd. (Wuhan, China) using a hybrid strategy combining Oxford Nanopore Technologies (ONT) long reads and Illumina short reads.

For ONT sequencing, a library was prepared with the Native Barcoding Kit V14 (SQK-NBD114.96, ONT, Oxford, UK) according to the manufacturer’s instructions and sequenced on a PromethION sequencer (ONT) using an R10.4.1 flow cell. For Illumina sequencing, a paired-end library was constructed following the TruSeq DNA Sample Preparation Guide (Illumina, 15,026,486 Rev. C) and sequenced on a NovaSeq X Plus platform (Illumina, San Diego, CA, USA) with paired-end 150 bp reads.

#### 2.7.2 Genome assembly and annotation

Hybrid *de novo* assembly was performed using Flye v2.9 (Kolmogorov et al., 2019), followed by polishing with Illumina reads using Pilon v1.24 (Walker et al., 2014). Plasmid detection was carried out using PlasFlow v1.1.0 (Krawczyk et al., 2018).

Gene prediction and structural annotation were carried out with Prokka v1.14.6 (Seemann, 2014). Genome completeness and contamination were assessed using CheckM v1.0.18 on the KBase platform, applying the lineage-specific workflow for the family Comamonadaceae (Parks et al., 2015).

Functional annotation was performed by aligning predicted protein sequences against the Kyoto Encyclopedia of Genes and Genomes (KEGG), Clusters of Orthologous Groups (COG), Non-Redundant Protein Database (NR), Gene Ontology (GO), InterPro, and Pfam databases. Biosynthetic gene clusters (BGCs) for secondary metabolites were predicted using antiSMASH v8.0.4 (Blin et al., 2025). Antimicrobial resistance genes were identified with the Resistance Gene Identifier (RGI, v6.0.5) by querying the Comprehensive Antibiotic Resistance Database (CARD, v4.0.1) under the *strict* parameter (Alcock et al., 2023). Virulence factors were screened using VFanalyzer against the Virulence Factor Database (VFDB) with default parameters (Liu et al., 2019). To detect potential virulence-related proteins, BLASTP searches were performed against the Pathogen-Host Interaction database (PHI-base, v4.17) with an E-value threshold of 1e-5 (Urban et al., 2025).

The raw sequence data of strain 24b have been deposited in the Genome Sequence Archive (GSA) at the National Genomics Data Center, China National Center for Bioinformation / Beijing Institute of Genomics, Chinese Academy of Sciences (GSA: CRA043597) that are publicly accessible at: https://ngdc.cncb.ac.cn/gsa.

#### 2.7.3 Taxonomic identification

The average nucleotide identity (ANI) between the genome of strain 24b and the genomes of all relevant type strains retrieved from the Type (Strain) Genome Server (TYGS) was calculated using the JSpecies Web Server (JSpeciesWS) with the BLAST+-based algorithm (ANIb) (Richter et al., 2015). Digital DNA–DNA hybridization (dDDH) values between strain 24b and the same set of reference strains were obtained from the TYGS platform^1^ under default settings (Meier-Kolthoff and Göker, 2019). A phylogenomic tree was also generated using TYGS, which employs the FastME algorithm for tree construction (Lefort et al., 2015). Species delineation thresholds of 95–96% for ANI and 70% for dDDH were applied (Meier-Kolthoff et al., 2013; Richter and Rosselló-Móra, 2009).

#### 2.7.4 Comparative genomic analysis of the delftibactin a/B biosynthetic gene cluster

A whole-genome phylogenetic tree was constructed using the CVTree4 webserver^2^ with default k-mer lengths of 5, 6, and 7. The draft genome of *Delftia acidovorans* Dac759 was included alongside other completed genomes. The outgroup species were *Delftia lacustris* FDAARGOS_890 (GCF_016027815.1) and *Delftia tsuruhatensis* TR1180 (GCF_009362995.1). Synteny comparison of the delftibactin A/B biosynthetic gene cluster (BGC) was compared using Clinker on the CAGECAT webserver^3^ with default settings. The order of strains in the synteny plot was manually adjusted to match the tree topology from the primary analysis (k = 6). The two figures were combined with Adobe Illustrator (v24.3). Reference genomes were downloaded from NCBI GenBank: RAY209 (CP029117.1), SPH-1 (CP000884.1), HK171 (CP018114.1), B804 (CP058970.1), and Dac759 (JBEELG000000000). The genome of strain 24b from this study was also included.

### 2.8 Statistical analysis

Statistical analyses were performed using SPSS (version 20.0, USA). Graphs were generated with Origin (version 2021, USA) and GraphPad Prism (version 11, USA). Data are presented as means ± standard deviation (SD) or means ± standard error of the mean (SEM) as indicated in the figure legends. For *in vivo* assays, each cultivation bag was treated as one biological replicate; multiple pilei within a bag were averaged to obtain a bag-level value. Normality was checked using the Shapiro–Wilk test, and homogeneity of variances was tested using Levene’s test. When both assumptions were met, parametric tests (one-way or two-way ANOVA, independent samples *t*-tests, and linear regression) were applied. Otherwise, non-parametric alternatives (Kruskal–Wallis with Dunn’s post-hoc) were used. Post-hoc tests are specified in the corresponding figure legends and Results sections. A significance level of *α* = 0.05 was adopted for all tests.

## 3 Results

### 3.1 Isolation and *in vitro* screening of antagonistic strains

**Figure 1:**
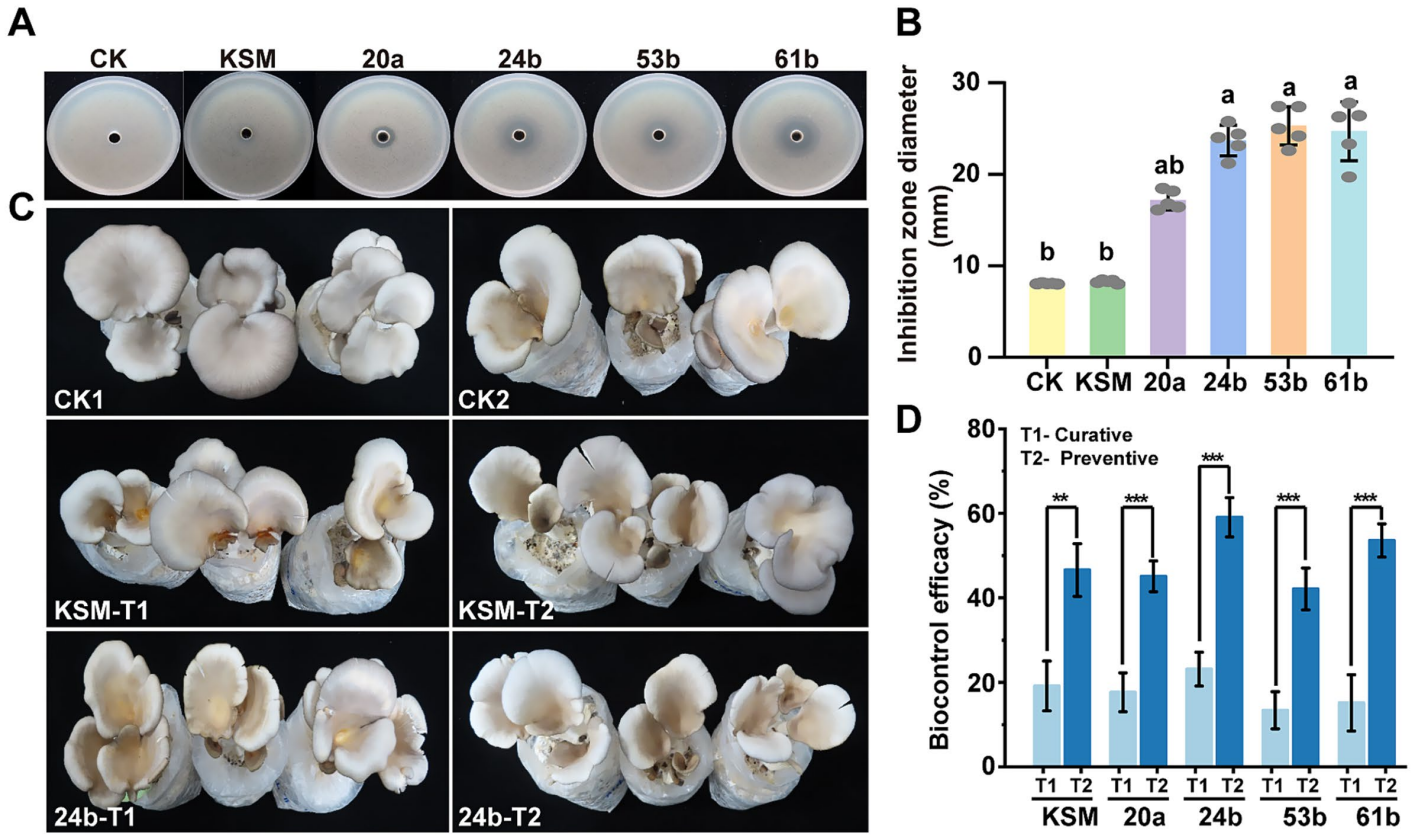
*In vitro* antagonistic activity and *in vivo* control efficacy of selected biocontrol strains. **(A)** Antagonistic effect against *Pseudomonas tolaasii* on PDA plates. **(B)** Diameters of inhibition zones (total diameter including the well; mean ± SD, *n* = 5). Kruskal–Wallistest (H = 24.96, df = 5, *p* = 0.0001) followed by Dunn’s post-hoc test with Bonferroni correction. Different letters above *n* = bars indicate significant differences (*p* < 0.05). CK: sterile water; KSM: 6% kasugamycin. **(C,D)** Biocontrol efficacy under curative (T1) and preventive (T2) application modes on cultivated *Pleurotus ostreatus* fruiting bodies. **(C)** Representative fruiting bodies. **(D)** Biocontrol efficacy (mean ± SEM, *n* = 10 bags). Normality (Shapiro–Wilk) and homogeneity of variances (Levene’s test) were confirmed. For each strain, an independent samples *t*-test was used to compare curative (T1) vs. preventive (T2) efficacy (***p < 0.001). Within each application mode, one-way ANOVA detected no significant differences among the five treatments (T1: *p* = 0.721; T2: *p* = 0.097); therefore no post-hoc comparisons were performed. CK1, sterile water control; CK2, pathogen-only control.

A total of 69 bacterial strains were isolated from *Pleurotus ostreatus* fruiting bodies with mild brown blotch disease. A two-step in vitro antagonistic assay identified four strains that produced stable inhibition zones against *Pseudomonas tolaasii* (Figure 1A). Strain 53b generated the largest inhibition zone (25.31 ± 2.08 mm), followed by strain 61b (24.70 ± 3.22 mm) and strain 24b (23.70 ± 1.68 mm), while strain 20a showed the smallest zone (17.18 ± 1.06 mm). Given the small sample size (*n* = 5 plates per strain), data were analyzed using the Kruskal–WallisH test followed by Dunn’s post-hoc test with Bonferroni correction. A significant difference was detected among the six treatment groups (H = 24.96, df = 5, *p* = 0.0001). No significant differences were detected among the four tested strains (*p* > 0.05), while all four strains produced significantly larger inhibition zones than the KSM and CK control (*p* < 0.05; Figure 1B).

### 3.2 *In vivo* screening of antagonistic strains under curative and preventive application modes

The selected strains and the positive control, kasugamycin (KSM), were evaluated under curative (T1: pathogen inoculation followed by biocontrol agent) and preventive (T2: biocontrol agent followed by pathogen inoculation) application modes on cultivated *P. ostreatus* fruiting bodies (Figure 1C). Prior to two-way ANOVA, the bag-level biocontrol efficacy data were assessed for normality (Shapiro–Wilk test) and homogeneity of variance (Levene’s test). Levene’s test indicated equal variances across the ten treatment groups (F_9, 90_ = 0.741, *p* = 0.670). Normality was satisfied for 8 of 10 groups (*p* > 0.05); minor deviations were observed only for T1-24b (*p* = 0.028) and T2-20a (*p* = 0.017). Given that ANOVA is robust to modest violations of normality with balanced sample sizes (*n* = 10), and that variance homogeneity was confirmed, parametric methods were retained. Two-way ANOVA revealed a highly significant main effect of application mode (F_1, 90_ = 100.093, *p* < 0.001), with estimated marginal mean control efficacy of 17.68 ± 2.23% for T1 and 49.28 ± 2.23% for T2. Neither the main effect of strain (F_4, 90_ = 1.936, *p* = 0.111) nor the interaction between application mode and strain (F_4, 90_ = 0.543, *p* = 0.704) reached statistical significance (Supplementary Table S1).

Within each application mode, one-way ANOVA revealed no significant differences among the five treatments (T1: *p* = 0.721; T2: *p* = 0.097). No post-hoc comparisons were performed because the overall ANOVA was not significant. Independent samples *t*-tests demonstrated that preventive efficacy was significantly higher than curative efficacy for all treatments (KSM: t_18_ = −3.201, *p* = 0.005; 20a: t_18_ = −4.476, *p* < 0.001; 24b: t_18_ = −5.857, *p* < 0.001; 53b: t_18_ = −4.334, *p* < 0.001; 61b: t_18_ = −4.969, *p* < 0.001; Figure 1D). Although no statistically significant differences were detected among strains, strain 24b consistently exhibited the highest mean efficacy under preventive application (59.06 ± 4.65%), which was 35.94 percentage points higher than its curative efficacy (23.12 ± 4.00%). Consequently, strain 24b was selected for further investigation and deposited at the China General Microbiological Culture Collection Center under the accession number CGMCC 34764.

### 3.3 Effect of application timing on control efficacy of strain 24b

**Figure 2:**
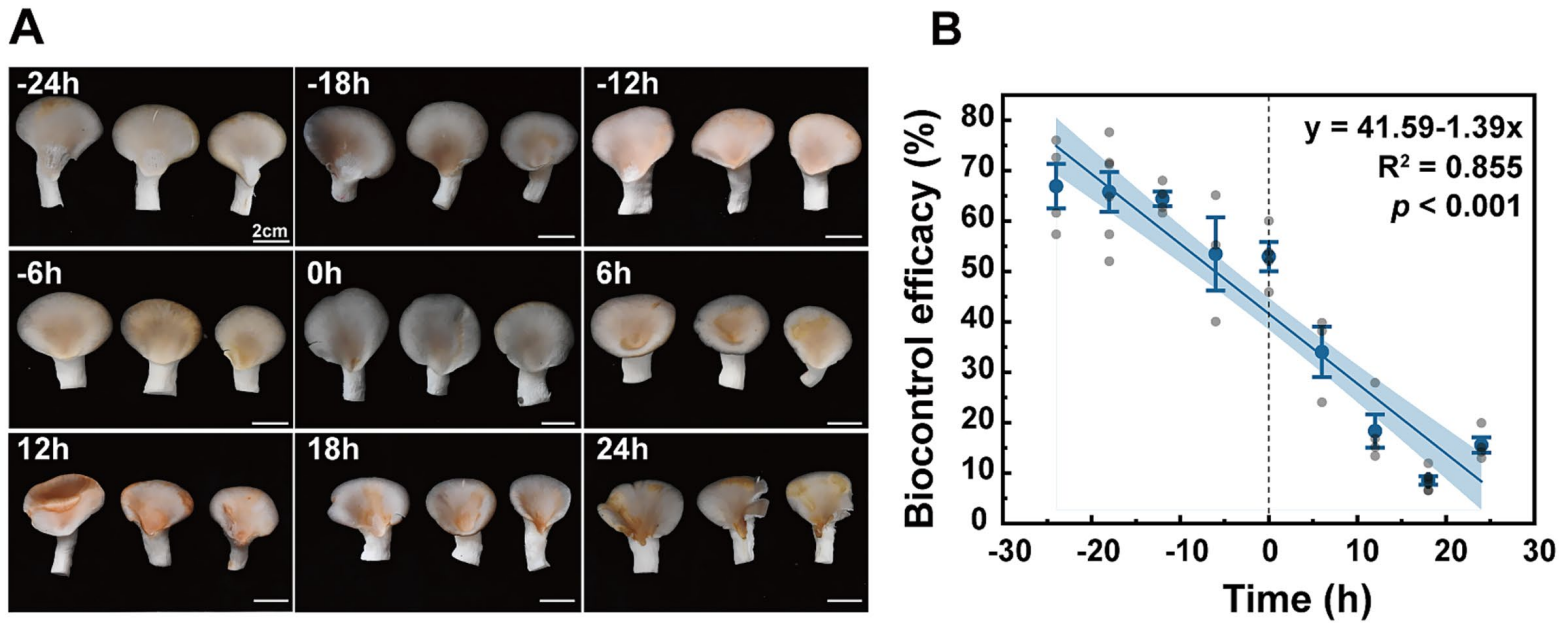
Effect of application timing on the control efficacy of strain 24b. **(A)** Disease phenotypes of *P. ostreatus* caps after application of biocontrol strain 24b at different time points ranging from 24 h before to 24 h after *P. tolaasii* inoculation. Scale bar = 2 cm. **(B)** Distribution of biocontrol efficacy data at each time point. Each translucent dot represents the biocontrol efficacy from a single cultivation bag. Sample sizes per time point (from −24 h to 24 h): 4, 6, 4, 3, 4, 3, 4, 6, 4; total *n* = 38. Kruskal–Wallistest revealed a significant effect of application timing (H = 32.63, df = 8, *p* < 0.0001) for Dunn’s post-hoc comparisons. Larger solid circles with vertical error bars represent means ± SEM (*n* = 3–6 bags per time point). The blue solid line represents the linear regression fit: *y* = 41.59–1.39×, with a coefficient of determination *R*^2^ = 0.855, *p* < 0.001. The light blue shaded area indicates the 95% confidence band of the regression. The dashed vertical line at Time = 0 h marks the co-inoculation of the pathogen and strain 24b.

Given that preventive application showed significantly higher efficacy than curative application, we next determined the optimal timing window by testing nine time points. Control efficacy was highest when strain 24b was applied 12–24 h before pathogen inoculation, reaching 64.4–66.9%. In contrast, post-inoculation application resulted in markedly lower efficacy, dropping to 34.0% at 6 h and below 20% at 12 to 24 h (Figure 2).

Sample sizes per time point were small (*n* = 3–6), so normality could not be reliably assessed. We therefore used a Kruskal–Wallistest to compare biocontrol efficacy across the nine time points. Application timing had a significant effect (H = 32.63, df = 8, *p* < 0.0001). Dunn’s post-hoc comparisons with Bonferroni correction revealed no significant differences among pre-inoculation timings (−24 h to 0 h; all *p* > 0.05). Control efficacies at −24 h and −18 h were significantly higher than those at 12 h, 18 h, and 24 h (*p* < 0.05), but were not significantly different from 6 h (*p* > 0.05; Supplementary Table S2). No other pairwise comparisons reached statistical significance (*p* > 0.05).

Linear regression using all individual data points (*n* = 38) demonstrated a strong negative relationship between application timing and control efficacy, described by the equation: Control efficacy (%) = 41.59–1.39 × Time (*R*^2^ = 0.855, *p* < 0.001; Figure 2B). A quadratic model did not significantly improve the fit (*R*^2^ = 0.865) and produced a biologically implausible optimum; therefore, the linear model was retained. The highest control efficacies were observed when strain 24b was applied 12–24 h before pathogen inoculation, while post-inoculation application was largely ineffective.

### 3.4 Compatibility and safety assessment of strain 24b with *P. ostreatus*

**Figure 3:**
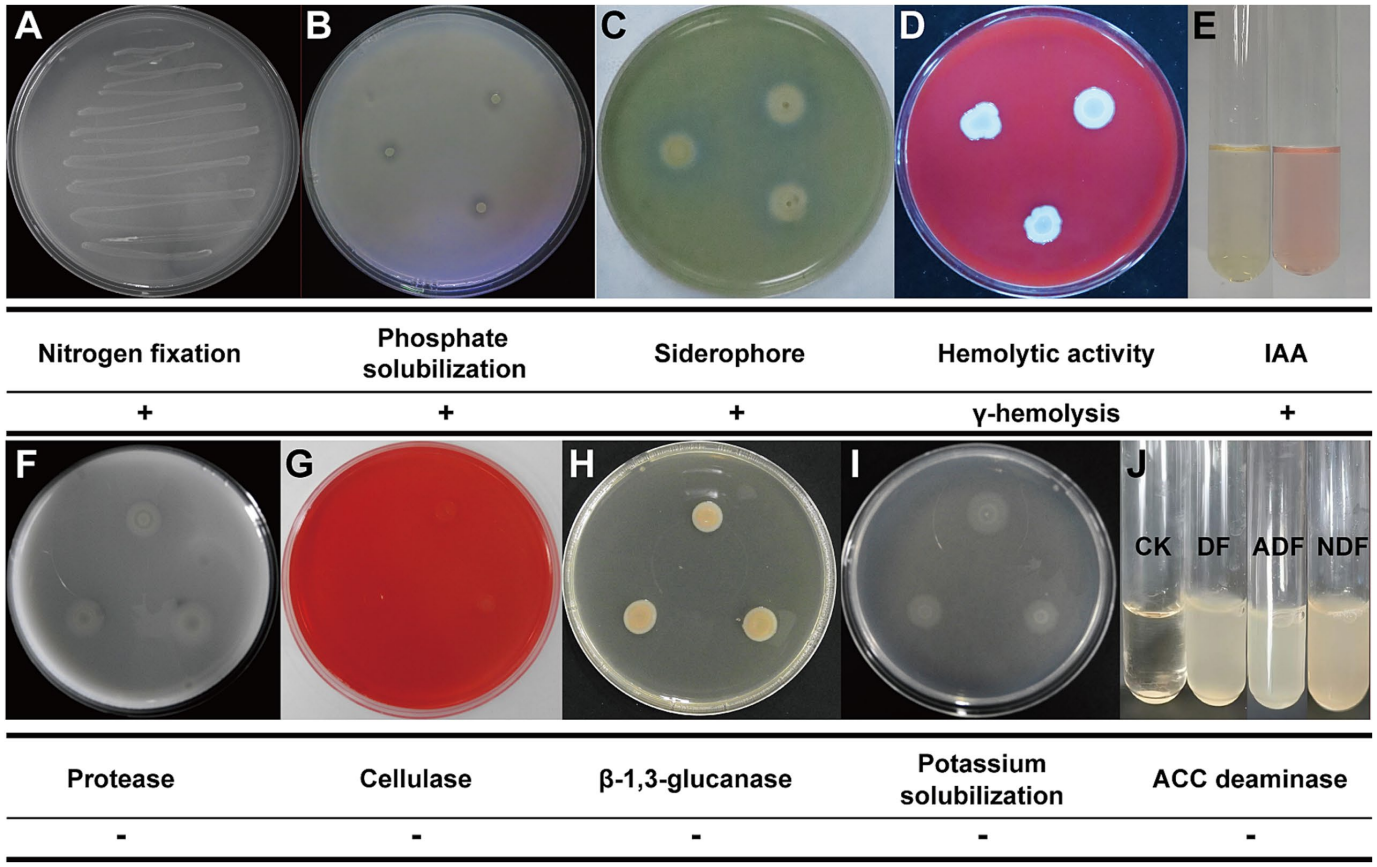
Biological control and plant growth-promoting traits of strain 24b. **(A)** Nitrogen fixation **(B)** Phosphate solubilization **(C)** Siderophore **(D)** Hemolytic activity **(E)** IAA generation **(F)** Protease **(G)** Cellulase **(H)** *β*-1,3-glucanase **(I)** Potassium solubilization **(J)** ACC deaminase; (+) and (−) indicate positive and negative results, respectively.

Phenotypic characterization revealed that strain 24b exhibits multiple mushroom growth-promoting (MGP) traits, demonstrating positive activities in atmospheric nitrogen fixation, inorganic phosphate solubilization, and indole-3-acetic acid (IAA) biosynthesis. It also produced siderophores (Figure 3C) but did not solubilize potassium or produce ACC deaminase. Extracellular enzymatic profiling revealed no protease, cellulase, or *β*-1,3-glucanase activities in this strain (Figure 3). Blood agar assays confirmed that strain 24b displays a strict *γ*-hemolytic (non-hemolytic) phenotype (Figure 3E).

**Figure 4:**
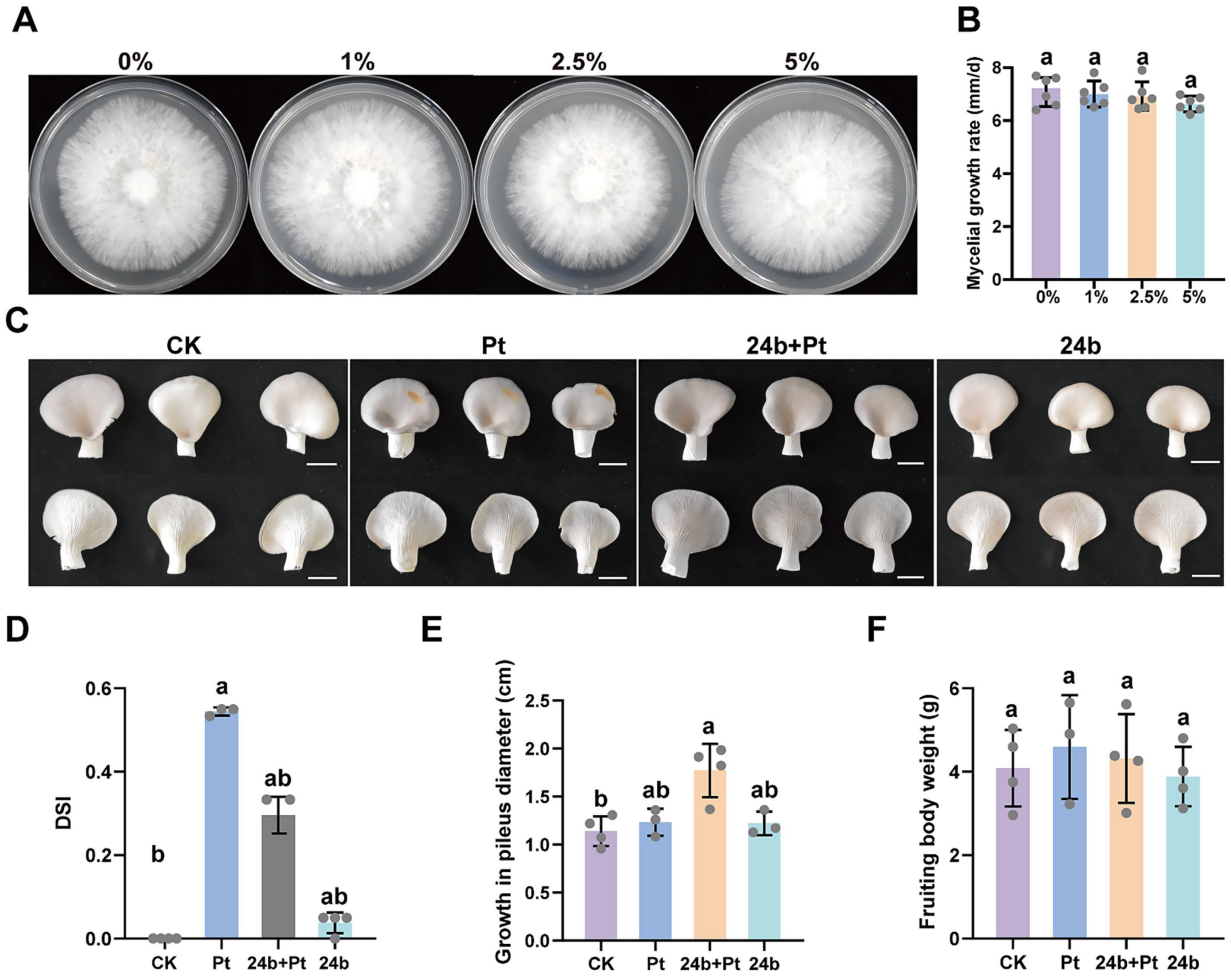
Compatibility and safety assessment of strain 24b with *P. ostreatus*. **(A)** Mycelial growth of *P. ostreatus* on PDA plates supplemented with 0, 1, 2.5, and 5% (v/v) cell-free culture filtrate of strain 24b after 7 days. **(B)** Mycelial growth rate (mm/d, mean ± SD, *n* = 6). **(C)** Representative photographs of fruiting bodies 24 h post-treatment. CK, negative control (sterile water); Pt, positive control (*P. tolaasii* only); 24b + Pt, preventive treatment (24b + *P. tolaasii*); 24b, safety treatment (24b only). **(D)** Disease severity index (DSI) of fruiting bodies. For DSI, each data point represents one cultivation bag (*n* = 3–4 cultivation bags per treatment) **(E)** Growth increment of pileus diameter (cm). **(F)** Fresh weight per fruiting body (g). Data are shown as mean ± SD (*n* = 3–4 bags per treatment); each translucent dot represents one cultivation bag. Different letters above bars indicate significant differences (Kruskal–Wallis test with Dunn’s post-hoc, *p* < 0.05).

The compatibility of strain 24b with *P. ostreatus* was evaluated at both the mycelial and fruiting body stages (Figure 4). On PDA plates, the addition of cell-free culture filtrate at concentrations up to 5% (v/v) did not affect mycelial growth. A Kruskal–Wallistest revealed no significant differences among the tested concentrations (H = 2.576, *p* = 0.4617; Figure 4B).

Regarding fruiting bodies, direct application of the strain 24b suspension caused no visible brown blotch symptoms. The disease severity index (DSI) differed significantly among treatments (Kruskal-Wallis, H = 13.06, *p* < 0.0001; Figure 4D). Pileus diameter increment also showed significant variation across treatments (Kruskal-Wallis, H = 8.595, *p* = 0.0130; Figure 4E). In contrast, fresh weight per fruiting body did not differ significantly among treatments (Kruskal-Wallis, H = 1.329, *p* = 0.7573; Figure 4F). Pairwise comparisons (Dunn’s post-hoc test) are indicated by different letters in Figures 4D,E.

### 3.5 Taxonomic identification of strain 24b

#### 3.5.1 Morphological and physiological characteristics

**Figure 5:**
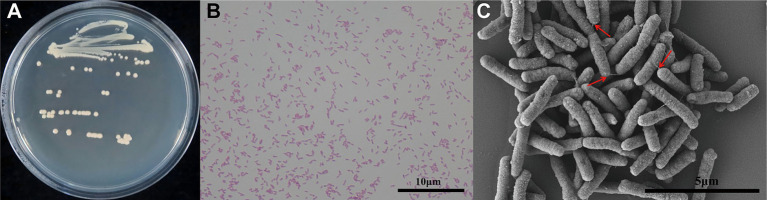
Morphology characterization of strain 24b. **(A)** Colony morphology of strain 24b on LB agar. **(B)** Gram staining of strain 24b (scale bar = 10 μm). **(C)** Scanning electron microscopy (SEM) image of strain 24b (scale bar = 5 μm); Arrows indicate flagella.

After incubation on Luria-Bertani (LB) medium at 28 °C for 48 h, colonies of strain 24b were round, convex, smooth, entire, and yellowish (Figure 5A). Gram staining showed strain 24b to be Gram-negative (Figure 5B). Scanning electron microscopy (SEM) revealed that the cells of strain 24b are short rod-shaped, measuring 1.8 to 4.2 × 0.65 to 0.76 μm, and possess a polar tuft of flagella (Figure 5C).

In physiological and biochemical tests, strain 24b utilized fructose, D-mannitol, citrate, malonate, and L-phenylalanine, but not D-glucose, D-xylose, or L-alanine. It hydrolyzed acetamide, produced indole, and tested positive for catalase and nitrate reduction. Negative results were observed for H₂S production, arginine dihydrolase, lysine decarboxylase, ornithine decarboxylase, starch hydrolysis, gelatin liquefaction, methyl red, Voges-Proskauer, oxidase, and lecithinase (Supplementary Table S3).

#### 3.5.2 Molecular identification and phylogenetic analysis

**Figure 6:**
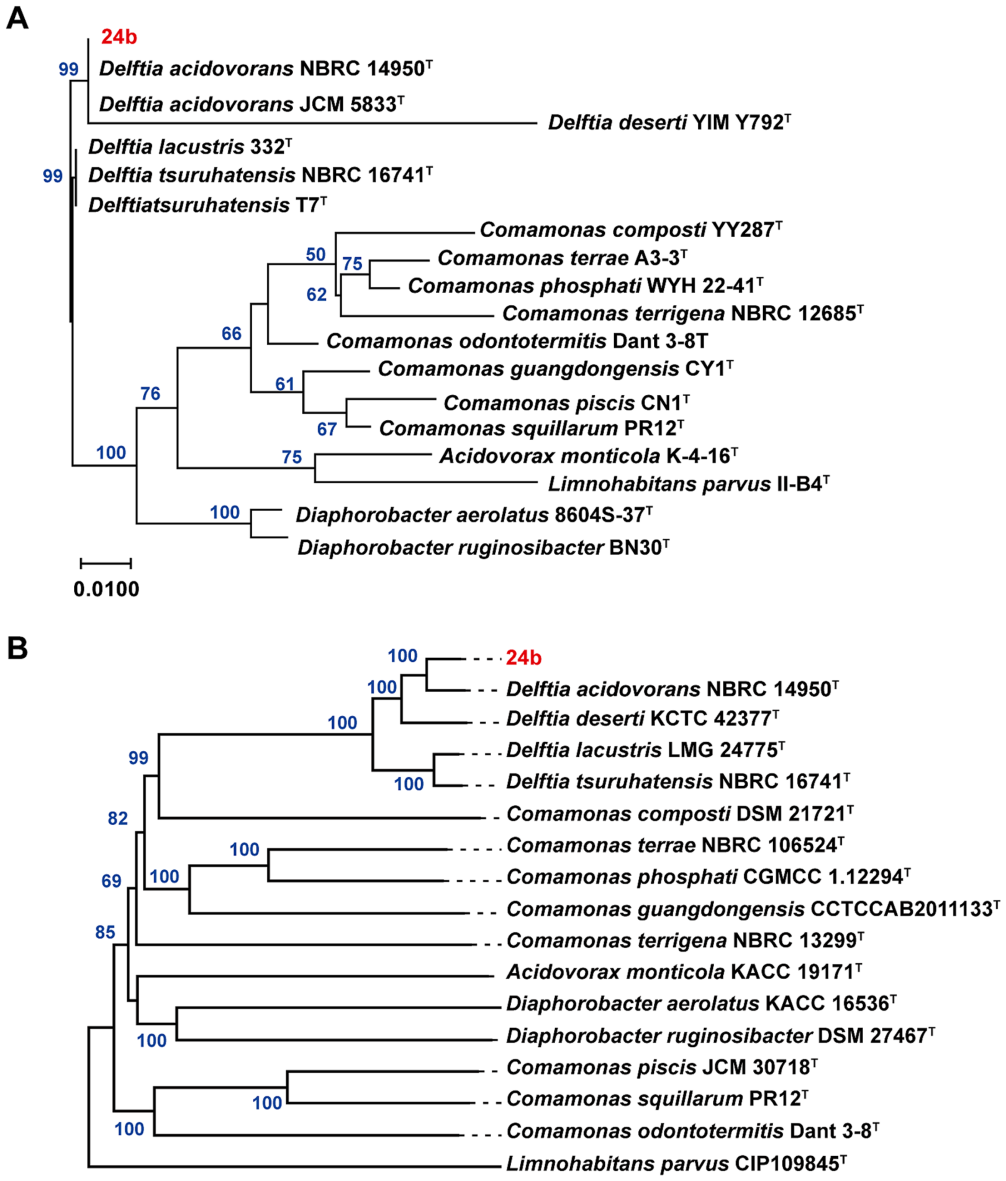
Phylogenetic relationships of strain 24b. **(A)** Maximum-likelihood phylogenetic tree based on 16S rRNA gene sequences; bootstrap values (1,000 replicates) are shown at the nodes, and the scale bar represents 0.01 substitutions per nucleotide position. **(B)** Phylogenomic tree inferred using TYGS, with Bootstrap values (1,000 replicates) shown at the nodes; type strains are marked with a superscript T.

The 16S rRNA gene of strain 24b was PCR-amplified and sequenced (GenBank accession no. PZ204722). BLASTn analysis showed 100% nucleotide identity to the type strain *D. acidovorans* NBRC 14950, and maximum-likelihood phylogenetic reconstruction placed strain 24b unambiguously within the *D. acidovorans* clade with high bootstrap support (Figure 6A).

Whole-genome comparisons confirmed this classification: genome-to-genome analyses with the type strain *D. acidovorans* NBRC 14950 yielded an ANI of 97.06% and a dDDH value of 80.10% (Supplementary Table S4), both exceeding the species delineation thresholds (≥ 95–96% for ANI and ≥ 70% for dDDH). Furthermore, phylogenomic analysis using the TYGS placed strain 24b in a well-supported monophyletic cluster with the type strain of *D. acidovorans* (Figure 6B). Thus, based on comprehensive polyphasic evidence, strain 24b is identified *as Delftia acidovorans*.

### 3.6 Whole-genome sequencing, assembly, and genomic features of strain 24b

Oxford Nanopore Technologies (ONT) sequencing yielded 1.21 Gb of clean data after filtering, with an N50 read length of 15,115 bp. Illumina sequencing produced 1.63 Gb of clean data, achieving a Q30 score of 95.60%. CheckM analysis, using the Comamonadaceae lineage marker set, indicated a genome completeness of 99.85% and a contamination rate of 0.26%. No plasmid sequences were detected.

**Figure 7:**
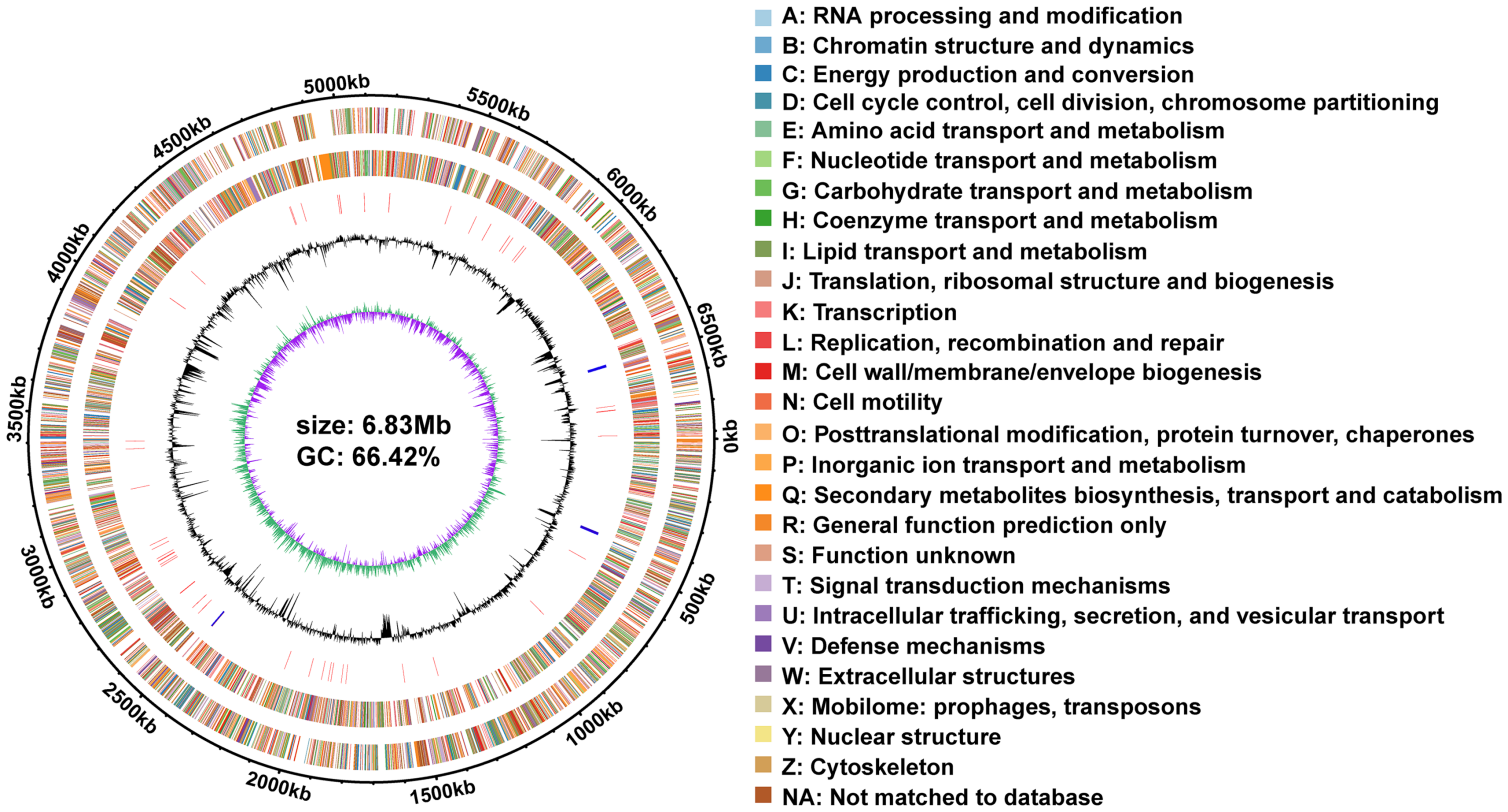
Circular genome map of strain 24b. The distribution of circles from outwards to inwards are: ring 1 for genome size and position; ring 2 and 3 for COG annotation of coding genes on forward and reverse strand, respectively; ring 4 for tRNAs (red) and rRNAs (blue); ring 5 for GC contents; ring 6 for GC skew value.

The complete genome of strain 24b consists of a single circular chromosome of 6,825,422 bp, with a GC content of 66.42% (Figure 7). It contains 6,156 genes, of which 6,012 are protein-coding sequences (CDSs) with an average length of 1,014 bp. The genome encodes five copies each of the 5S, 16S, and 23S rRNA genes, with average lengths of 107 bp, 1,529 bp, and 2,873 bp, respectively. Additionally, it encodes 84 tRNAs (average length, 79 bp), two tmRNAs (average length, 390 bp), and 43 miscellaneous RNAs (Supplementary Table S5).

Functional annotation assigned 5,997 of the 6,012 CDSs (99.75%) to at least one public database. Among these, 5,996 genes (99.73%) were annotated in NR, 5,091 (84.68%) in COG, 4,770 (79.34%) in KEGG, and 4,500 (74.85%) in GO (Supplementary Table S6).

### 3.7 Putative antimicrobial resistance and virulence-related genes identified in strain 24b

A search against the CARD database using RGI *strict* mode identified a single putative resistance gene, *adeF,* with 70.7% amino acid identity to an RND-type efflux pump. No other clinically relevant antimicrobial resistance genes were detected. Additional matches with lower identity (42.6–58.0%) fell below the confidence threshold and were therefore not considered significant (Supplementary Table S7).

VFanalyzer screening against the VFDB database using stringent thresholds (≥80% amino acid identity and ≥60% query coverage) detected no known virulence factors. However, when less stringent criteria (≥40% identity and ≥60% coverage) were applied, several low-similarity hits were identified. The most abundant VFDB categories included nutritional/metabolic factors and immune modulation, followed by effector delivery systems and adherence (Supplementary Figure S2A).

The Pathogen-Host Interaction (PHI) database was queried using BLASTP with an E-value threshold of ≤ 1e-5. Most hits were annotated by PHI-base as “reduced virulence,” while smaller proportions were classified as “unaffected pathogenicity,” “increased pathogenicity,” and “loss of pathogenicity” (Supplementary Figure S2B). A Venn diagram revealed that 307 genes were unique to VFDB, 1,509 were unique to PHI-base, and 1,005 were shared between the two databases (Supplementary Figure S2C). Analysis of sequence identity distributions showed that CARD hits had the highest median identity, around 57%, whereas VFDB and PHI-base hits had lower medians of approximately 45 and 40%, respectively (Supplementary Figure S2D).

### 3.8 Analysis of biosynthetic gene clusters in strain 24b

Biosynthetic gene clusters (BGCs) were predicted using antiSMASH version 8.0.4. In total, five BGCs were identified within the genome of strain 24b, comprising clusters responsible for the biosynthesis of resorcinol, two terpene-related compounds, a RiPP-like peptide, and an NRPS-T1PKS metallophore (Table 1, Supplementary Figure S3). Notably, Cluster 5 demonstrated a high similarity score (1.40) to the delftibactin A/B biosynthetic gene cluster (MIBiG accession BGC0000984.4). The remaining four clusters exhibited lower similarity scores, ranging from 0.41 to 0.51, corresponding to known clusters for bartoloside E-K, astallatene, zeaxanthin, and aeruginosamide B/C, respectively (Table 1).

**Table 1:** Summary of biosynthetic gene clusters identified in 24b.

Cluster	Type	Length (bp)	Most similar known cluster	Similarity confidence	Similarity score	MIBiG comparison compounds	MIBiG accession
1	Resorcinol	41,953	–	–	0.41	Bartoloside E-K	BGC0001526.4
2	Terpene-precursor	20,951	–	–	0.47	Astallatene	BGC0002397.2
3	Terpene	21,798	–	–	0.51	Zeaxanthin	BGC0000656.5
4	RiPP-like	13,791	–	–	0.41	Aeruginosamide B/C	BGC0000483.5
5	NRP-metallophore, NRPS, T1PKS	91,815	delftibactin A/B	High	1.40	Delftibactin A/B	BGC0000984.4

RiPP, ribosomally synthesized and post-translationally modified peptide; NRPS, non-ribosomal peptide synthetase; PKS, polyketide synthase. Similarity score is a composite score from antiSMASH MIBiG comparison; higher scores indicate greater overall genetic similarity.

### 3.9 Comparative analysis of the delftibactin a/B biosynthetic gene cluster

**Figure 8:**
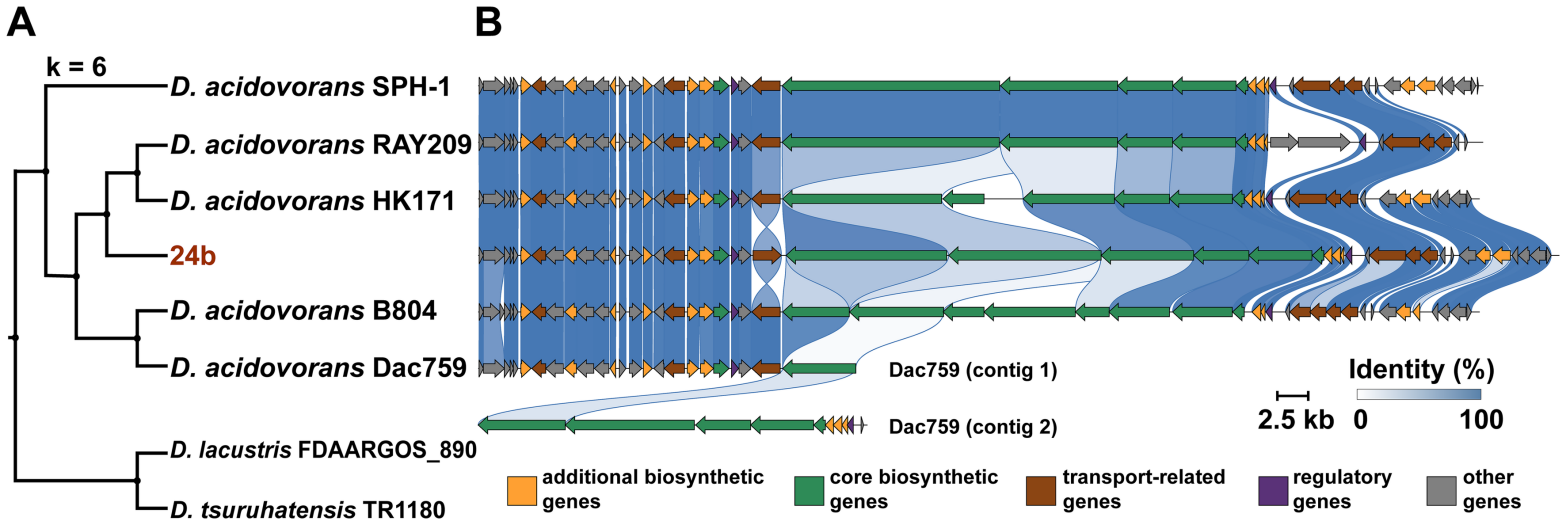
Phylogenetic relationships and comparative analysis of delftibactin A/B biosynthetic gene cluster (BGC) among *Delftia acidovorans* strains. **(A)** Whole-genome-based phylogenetic tree constructed using CVTree4 (k = 6). **(B)** Synteny of the delftibactin A/B BGC generated with clinker. Arrows represent genes, colour-coded by function: green, core biosynthetic; orange, additional biosynthetic; purple, regulatory; brown, transport-related; grey, other. Blue ribbons indicate homologous segments, with colour intensity proportional to nucleotide identity (0–100%). The scale bar represents 2.5 kb. Strain Dac759 is shown in two rows because the BGC is split across two contigs in the current genome assembly.

A whole-genome phylogenetic tree was constructed using CVTree4 with a k-mer size of 6 (Figure 8A). The outgroup species, *Delftia lacustris* FDAARGOS_890 and *Delftia tsuruhatensis* TR1180, were positioned outside the *Delftia acidovorans* clade. Within the *D. acidovorans* clade, strain SPH-1 formed a distinct, solitary branch. The remaining five strains (24b, RAY209, HK171, B804, Dac759) constituted a separate branch, in which B804 and Dac759 clustered closely together, while strain 24b formed an independent branch distinct from the RAY209 and HK171 cluster.

Pairwise genetic distances derived from the same k-mer analysis are presented in Supplementary Figure S8 (lower triangular matrix). The genetic distance between strains 24b and Dac759 was 0.105. Distances from strain 24b to HK171, RAY209, SPH-1, and B804 were 0.115, 0.117, 0.135, and 0.137, respectively. Among the plant-beneficial strains RAY209, SPH-1, and HK171, distances ranged from 0.084 to 0.135. All distances between the *D. acidovorans* group and the two outgroup species exceeded 0.178.

The synteny of the delftibactin A/B biosynthetic gene cluster was compared using clinker (Figure 8B). All six strains shared a conserved core region comprising biosynthetic, regulatory, and transport genes, with an identical gene order. In strain Dac759, the cluster was fragmented across two contigs due to an incomplete genome assembly. The clinical isolate B804 showed minor differences in the flanking regions compared with strains RAY209 and SPH-1. No major gene loss or rearrangement was observed in strain 24b relative to RAY209 or SPH-1.

## 4 Discussion

In this study, *Delftia acidovorans* strain 24b was isolated from the surface of mildly diseased *Pleurotus ostreatus* fruiting bodies affected by brown blotch disease, primarily caused by *Pseudomonas tolaasii*. The strain demonstrated consistent antagonistic activity *in vitro* and significantly decreased disease severity when applied preventively prior to pathogen inoculation in bag cultivation experiments. To the best of our knowledge, this represents the first report of a *Delftia* species functioning as a biocontrol agent against a bacterial pathogen of edible fungi.

Several antagonistic bacteria have been reported to inhibit *P. tolaasii*. Fermor and Lynch (1988) isolated fluorescent pseudomonads from mushroom farms and demonstrated stable antagonistic activity on excised tissue blocks. Tajalipour et al. (2014) identified *Pseudomonas putida*, *P. fluorescens,* and *Bacillus subtilis* strains, with a *P. fluorescens* strain reaching nearly 100% inhibition on detached caps when applied preventively. Aslani et al. (2018) isolated endophytic *Pseudomonas* and *Bacillus* strains from wild mushrooms, resulting in reductions of brown blotch symptoms by 96.7 and 72.25%, respectively, on excised tissue blocks. TSUKAMOTO et al. (1998, 2014) and Hermenau et al. (2020) investigated tolaasin detoxification, revealing that *Mycetocola* species linearize the cyclic lipodepsipeptide via lactonases, thereby neutralizing pathogen virulence. Collectively, these studies have established a significant foundation for the biocontrol of brown blotch disease.

The majority of high efficacy values reported to date have been derived from detached mushroom tissue blocks or small-scale laboratory assays; however, validation under actual production conditions remains limited. In contrast, we assessed strain 24b using bag cultivation experiments that more accurately replicate commercial mushroom production. Although the preventive efficacy of strain 24b is lower than some *in vitro* or excised-tissue results, it was evaluated on intact fruiting bodies under conditions that simulate natural pathogen pressure and encompass the entire cropping cycle, thereby rendering the findings more directly applicable to field conditions. Importantly, this study introduces the genus *Delftia* as a biocontrol agent against brown blotch disease for the first time and provides a complete delftibactin A/B biosynthetic gene cluster as a molecular resource. This discovery not only broadens the repertoire of bacterial genera with documented biocontrol potential but also offers a novel avenue for the development of antibacterial metabolites and the elucidation of a previously uncharacterized antagonistic mechanism.

Strain 24b was also screened for mushroom growth-promoting (MGP) traits, including nitrogen fixation, phosphate solubilization, and indole-3-acetic acid (IAA) production, because these properties are routinely examined in mushroom-associated bacteria and have been linked to improved yield (Kumari and Naraian, 2021; Paul et al., 2023; Young et al., 2013). The strain tested positive for all three activities in vitro. However, neither cell-free culture filtrate (up to 5% v/v) nor direct bacterial application affected mycelial growth or fruiting body development of *P. ostreatus*. Bacterial isolates from mushroom substrates often test positive for MGP traits in vitro but fail to promote growth under cultivation conditions. The absence of any growth-promoting effect in strain 24b helps rule out host growth enhancement as a mechanism of disease suppression, thereby attributing the observed biocontrol efficacy to direct antagonistic activity against *P. tolaasii*.

The efficacy of control measures was highly dependent on the timing of application. Preventive treatments yielded an average efficacy of 49.28%, while curative applications achieved only 17.68%. The optimal application window was identified as 12 to 24 h prior to inoculation, with efficacy ranging from 64.4 to 66.9%. A linear regression analysis (*R*^2^ = 0.855) demonstrated a strong negative correlation between application timing and control efficacy. This time-dependent effect cannot be solely attributed to direct antibiosis, suggesting the involvement of microbe-induced resistance mechanisms. In plants, certain non-pathogenic bacteria are known to prime the immune system, resulting in more rapid and robust defense responses upon subsequent pathogen challenge (Pieterse et al., 2014). Comparable phenomena have been reported in the *P. ostreatu*s–*P. tolaasii* pathosystem. Zhao et al. (2013) isolated non-pathogenic bacterial strains from *Pleurotus* spp. and identified three strains capable of inducing resistance against *P. tolaasii*, achieving up to 66.7% control efficacy in field trials. Similarly, Xu (2013) screened 363 bacterial isolates and identified a weakly virulent strain of *P. tolaasii* which, after inactivation, induced systemic resistance in *P. ostreatus* at an optimal concentration of 3 × 10^8^ CFU/mL with a 24 h induction interval; three induction cycles resulted in a maximum protection rate of 80.1%. These findings indicate that *P. ostreatus* possesses an inducible defense system that can be activated by appropriate microbial elicitors. In the present study, pre-treatment with strain 24b may have triggered defense responses in *P. ostreatus* fruiting bodies, such as reinforcement of the epidermal cell wall, accumulation of reactive oxygen species, or enhanced production of antimicrobial metabolites.

Whole-genome sequencing analysis revealed that strain 24b harbors a complete delftibactin A/B biosynthetic gene cluster (Cluster 5, similarity score 1.40; Table 1), which encodes a non-ribosomal peptide siderophore potentially involved in multiple aspects of the biocontrol phenotype. Delftibactin A has been reported to exhibit broad-spectrum antimicrobial activity against multidrug-resistant bacteria, including methicillin-resistant *Staphylococcus aureus* (MRSA), vancomycin-resistant *Enterococcus* (VRE), and *Acinetobacter baumannii.* This activity is competitively inhibited by excess iron, indicating that its iron-chelating properties are closely linked to its antimicrobial function (Tejman-Yarden et al., 2019). In mushroom cultivation substrates, iron often represents a key limiting nutrient in microbial competition. Siderophores efficiently chelate environmental iron; thus, by secreting siderophores, strain 24b competes with *P. tolaasii* for this limited resource, thereby suppressing pathogen growth without exerting direct bactericidal effects (Loper and Henkels, 1999). Recent studies have confirmed that siderophore-mediated iron deprivation constitutes a core mechanism by which rhizosphere and phyllosphere bacteria inhibit *Ralstonia solanacearum* and fungal pathogens across diverse plant-pathogen systems (Gu et al., 2020; Zheng et al., 2025). Beyond nutrient competition, siderophores can function as “Trojan horses” by facilitating the transport of non-iron toxic metal ions or conjugated antibiotics into pathogen cells, thereby disrupting their iron-dependent metabolism (Kircheva et al., 2024). The theoretical foundation of this strategy and its clinical translation, exemplified by the successful application of the siderophore-cephalosporin conjugate drug Cefiderocol, provide supporting evidence for the potential antimicrobial mechanism associated with the delftibactin gene cluster. However, direct experimental data regarding the independent antimicrobial activity of delftibactin B remain limited, and its functional role requires further validation. In addition to Cluster 5, antiSMASH analysis predicted four additional secondary metabolite biosynthetic gene clusters with low similarity scores (Table 1). Although the specific contributions of these clusters to the biocontrol phenotype remain unclear, the presence of multiple biosynthetic gene clusters suggests that strain 24b possesses the genetic capacity to produce several antimicrobial secondary metabolites, which may synergistically enhance its inhibitory effects against the brown blotch pathogen through mechanisms involving iron competition and active metabolite production.

Safety assessment in this study addressed two distinct dimensions: compatibility with the host (*P. ostreatus*) and potential risk to human consumers. Regarding host compatibility, strain 24b showed no detectable cellulase or *β*-1,3-glucanase activities and did not induce lesions on fruiting bodies, confirming that it does not damage the fungal host. Concerning human safety, strain 24b exhibited a non-hemolytic phenotype and lacks known virulence factors. *D. acidovorans* is classified as Risk Group 1/BSL-1 by DSMZ and ATCC, indicating a low risk to healthy individuals. Consistent with this classification, Da Silveira et al. (2019) applied *D. acidovorans* directly to sugarcane, a food crop, and observed no adverse effects. Clinically, *D. acidovorans* infections are extremely rare and occur almost exclusively in immunocompromised patients; no foodborne outbreaks have been attributed to this species (Højgaard et al., 2022; Scaglione et al., 2025). Moreover, *P. ostreatus* is almost always consumed cooked, which inactivates vegetative bacteria. Further safety studies, such as acute oral toxicity and allergenicity tests, are needed before direct food application.

Phylogenetic analysis positioned strain 24b alongside the plant-beneficial strains RAY209, SPH-1, and HK171, distinctly separated from the clinical isolate B804 and the colistin-resistant isolate Dac759 (Figure 8A). Synteny analysis of the delftibactin BGC revealed conserved core regions across all strains, with the genomic structure of strain 24b matching that of the beneficial strains (Figure 8B). Strain 24b exhibited a *γ*-hemolytic (non-hemolytic) phenotype (Figure 3D), and screening against the VFDB detected no known virulence factors. The CARD analysis detected only an intrinsic efflux pump gene, *adeF,* sharing 70.7% identity with an RND-type efflux pump (Supplementary Figure S7); this gene is commonly associated with intrinsic resistance in environmental bacteria and does not indicate acquired drug resistance. Although the PHI-base search yielded several hits at lower identity thresholds, none met the ≥80% identity criterion, and most corresponded to basic metabolic genes and are not considered specific virulence determinants (Supplementary Figure S2). Direct compatibility assays demonstrated that cell-free culture filtrate at concentrations ≤5% did not inhibit mycelial growth, and application of bacterial suspensions to fruiting bodies did not induce lesions. Notably, strain 24b did not produce cellulase or β-1,3-glucanase, two enzymes that degrade key structural components of the fungal cell wall, indicating that it cannot cause structural damage to the host mycelium. Collectively, these findings confirm that strain 24b is safe for *P. ostreatus* and exhibits characteristic traits of a mushroom growth-promoting bacterium.

The selection of mildly diseased fruiting bodies as the isolation source was based on the observation that these tissues had naturally restricted pathogen progression, implying that their surface microbiota might be enriched with host-associated defensive strains. This ecological approach aligns with the concept of “host-endogenous defensive microbiota” (Carvalhais et al., 2017; Pieterse et al., 2014). Previous studies have isolated antagonistic bacteria from healthy mushroom tissues (Jafaripour et al., 2024) or screened for antagonists from compost and casing soil (Tajalipour et al., 2014). However, these studies did not specifically focus on “mildly diseased” tissues characterized by naturally limited pathogen spread, nor did they explicitly hypothesize that such tissues could serve as reservoirs of antagonistic bacteria. In contrast, the present study systematically targeted the surface microbiota of mildly diseased *P. ostreatus* fruiting bodies and successfully isolated a *Delftia* strain, a genus not previously reported in the biocontrol of edible mushroom diseases. This finding validates the feasibility of using mildly diseased tissues as a source of host-adapted antagonists and introduces a novel strategy for identifying biocontrol agents in edible mushroom cultivation.

Several limitations of this study should be noted. First, the experiments were conducted under controlled laboratory and semi-controlled greenhouse conditions; therefore, the results may differ under commercial production settings, and further pilot-scale evaluation is needed. Second, the specific antimicrobial compounds responsible for directly inhibiting *P. tolaasii* were not isolated or purified, and the genomic predictions require chemical confirmation. Third, the evidence for induced resistance is indirect, based mainly on the timing of application; direct measurement of defense-related gene expression or metabolite changes would strengthen this conclusion. Fourth, colonization was not directly measured. The high preventive efficacy only indirectly suggests effective colonization, but direct evidence (e.g., GFP-tagged strains, quantitative real-time PCR, strain re-isolation, or microbial community analysis) is needed to confirm the proposed niche-competition mechanism. Additionally, although strain 24b was isolated from surface-sterilized fruiting bodies, we did not verify sterilization effectiveness by plating the final rinse solution; thus, its endophytic status remains unconfirmed. The strain is therefore described throughout as a mushroom-associated bacterium.

Future research should prioritize the following areas: (i) elucidation of the molecular mechanisms underlying induced resistance through transcriptomic analyses; (ii) identification and characterization of bioactive compounds using liquid chromatography-mass spectrometry (LC–MS); (iii) comparative genomic studies involving other *Delftia* strains; (iv) direct observation of colonization dynamics on the surface of mushrooms. Despite the limitations noted, this study addresses a significant knowledge gap concerning *Delftia* in the biocontrol of edible mushrooms and offers a novel bacterial resource for the sustainable management of brown blotch disease.

## 5 Conclusion

*Delftia acidovorans* strain 24b was isolated from the surface of mildly diseased *Pleurotus ostreatus* fruiting bodies. This strain demonstrated inhibitory effects against *Pseudomonas tolaasii in vitro* and significantly reduced the severity of brown blotch disease when applied preventively 12 to 24 h prior to pathogen inoculation. Whole-genome sequencing revealed a complete delftibactin A/B biosynthetic gene cluster, which may contribute to antagonistic activity via siderophore-mediated iron competition or direct antimicrobial effects. Phylogenetic analysis positioned strain 24b within the plant-beneficial *Delftia* clade, and synteny comparisons indicated that its delftibactin biosynthetic gene cluster closely resembles those of previously characterized beneficial strains. Safety assessments revealed no hemolytic activity and no known virulence factors. To our knowledge, this study represents the first report of a *Delftia* strain capable of controlling a bacterial disease of edible fungi. The strain exhibits potential for development as an environmentally friendly biocontrol agent. Furthermore, the isolation strategy, which targets mildly symptomatic host tissues, may provide a generalizable approach for identifying host-adapted antagonists in edible mushroom cultivation.

## Data Availability

The genome sequence of Delftia acidovorans strain 24b has been deposited in the NCBI GenBank database and is currently under processing. The BioProject accession number is PRJNA1443980 (BioSample: SAMN56735050). The raw sequence data reported in this paper have also been deposited in the Genome Sequence Archive at the National Genomics Data Center (Nucleic Acids Res 2025), China National Center for Bioinformation/Beijing Institute of Genomics, Chinese Academy of Sciences, under the accession number GSA: CRA043597, and are publicly accessible at https://ngdc.cncb.ac.cn/gsa.

## Footnotes

1
https://tygs.dsmz.de

2
https://cvtree.online/v4/prok/

3
https://cagecat.bioinformatics.nl/tools/clinker
