# Supplementary material for: Preventive application of Delftia acidovorans suppresses Pseudomonas tolaasii-induced brown blotch in Pleurotus ostreatus
Source: Front Microbiol. 2026 Jun 30;17:1881575. doi: 10.3389/fmicb.2026.1881575 (PMC13364761; doi:10.3389/fmicb.2026.1881575)
Supplement: Supplementary file 2 [file Table_2.DOCX]

**
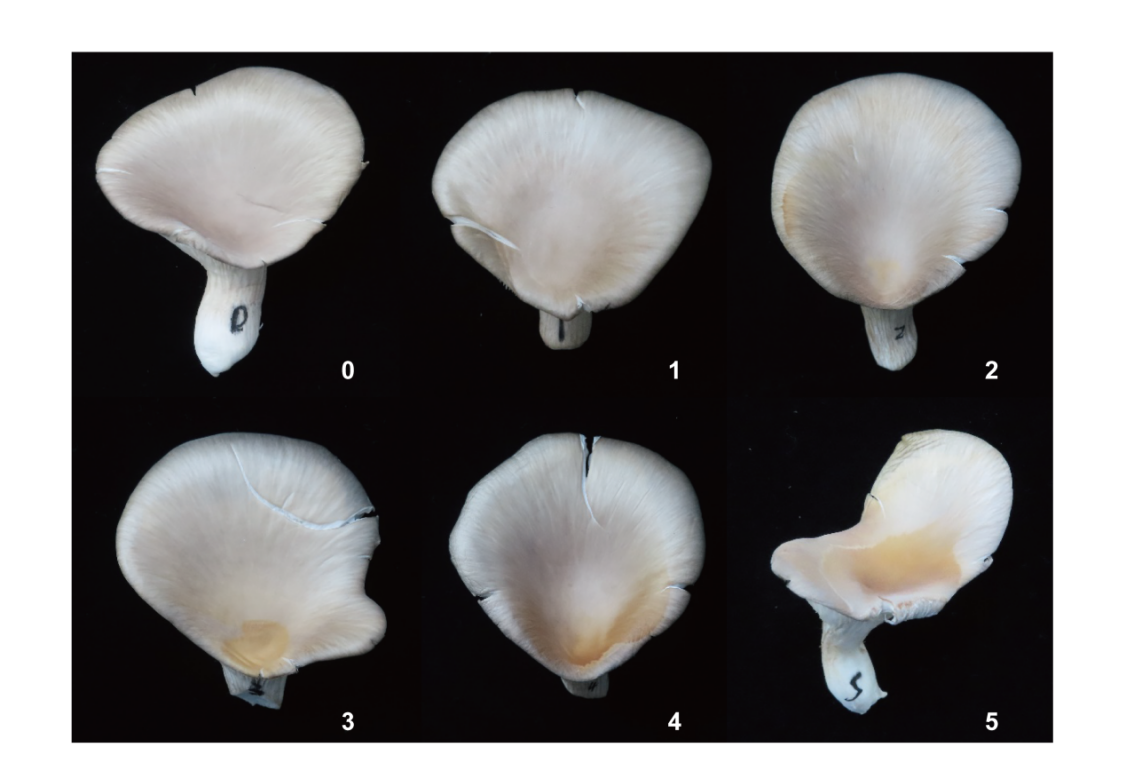
**

**Supplementary Figure S1** **Disease severity scale of *P. ostreatus* brown blotch caused by *P. tolaasii*.** 0: no lesions; 1: slight tissue discoloration, or water-soaked appearance, and no yellow lesions; 2: obvious yellowing of the pileus, flat surface without depression; 3: scattered punctate sunken lesions, lesions not coalescing; 4: confluent sunken lesions, affected area <50%, with few or no bacterial exudate; 5: large yellowish‑brown sunken lesions, affected area ≥50%, with obvious bacterial exudate.**
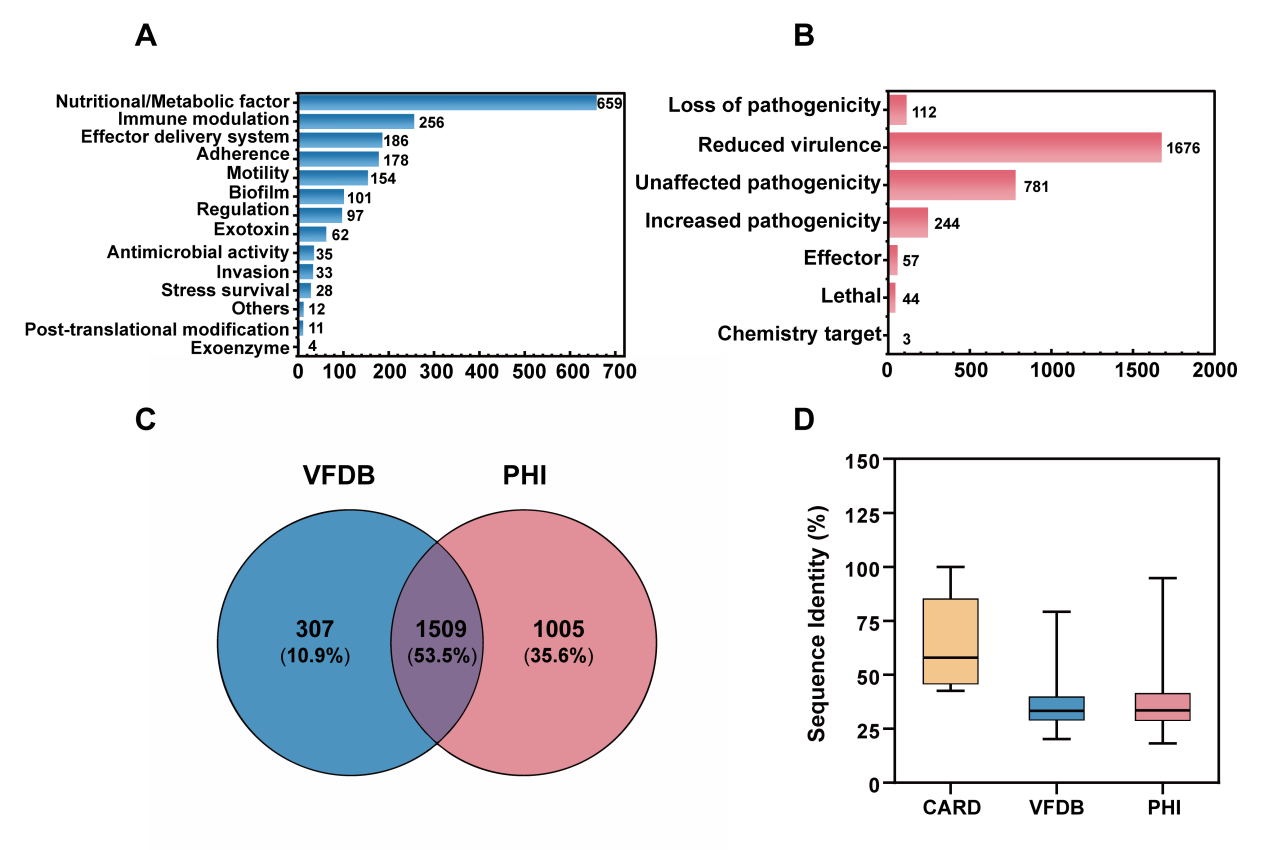
**

**Supplementary Figure S2 Genomic profiling of virulence and pathogenicity-associated factors in strain 24b.** (A) Functional categories of genes annotated against the Virulence Factor Database (VFDB). Bars represent the number of unique genes assigned to each category. (B) Mutant phenotypes of genes annotated against the Pathogen-Host Interactions (PHI) database. Bars represent the number of unique genes assigned to each phenotype. (C) Overlap of gene sets annotated by VFDB and PHI. Numbers indicate unique and shared genes between the two databases. (D) Sequence identity (Pident) of BLASTP matches to VFDB (n = 1,816), PHI (n = 2,917), and CARD (n = 4) databases. Boxes indicate interquartile range (IQR), horizontal lines denote medians, and whiskers extend to 1.5 × IQR.

**
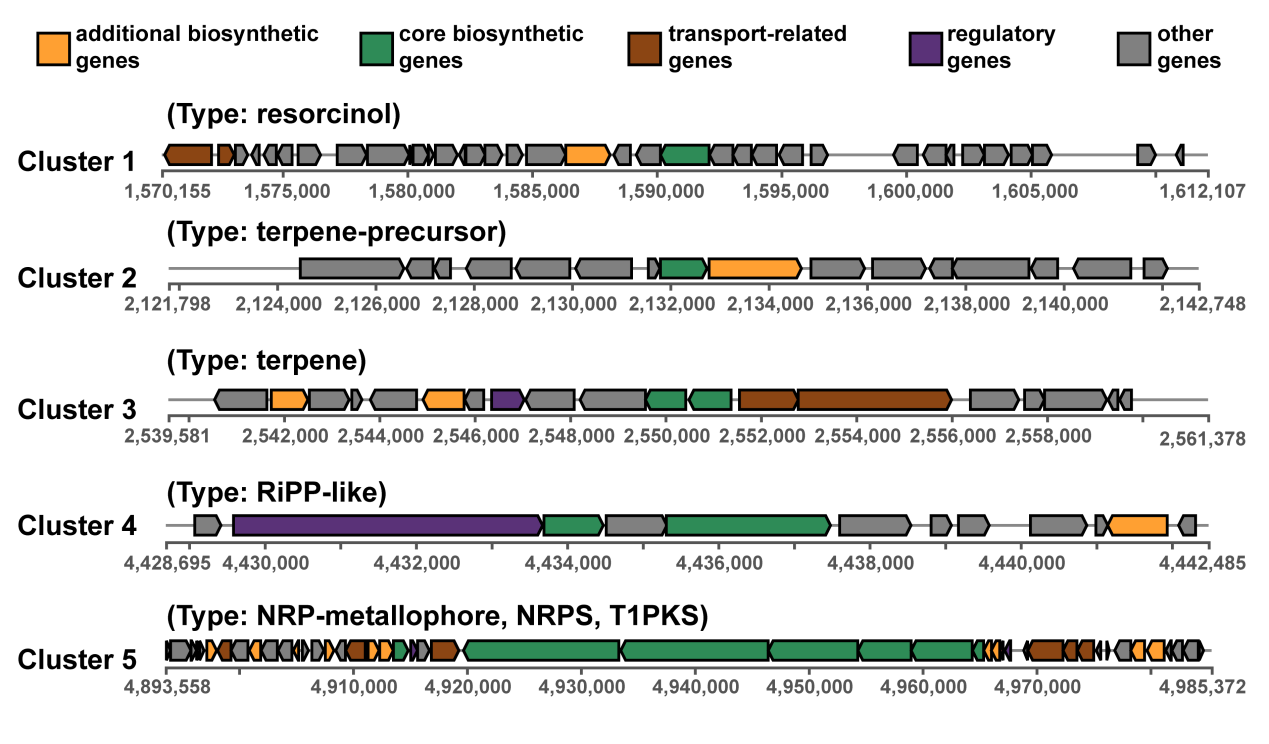
**

**Supplementary Figure S3 BGCs of strain 24b genome predicted by antiSMASH (version 8.0.4).** Colored arrows indicate gene functions: orange, additional biosynthetic genes; green, core biosynthetic genes; brown, transport-related genes; purple, regulatory genes; gray, other genes. Cluster lengths are shown in nucleotides (nt).

**
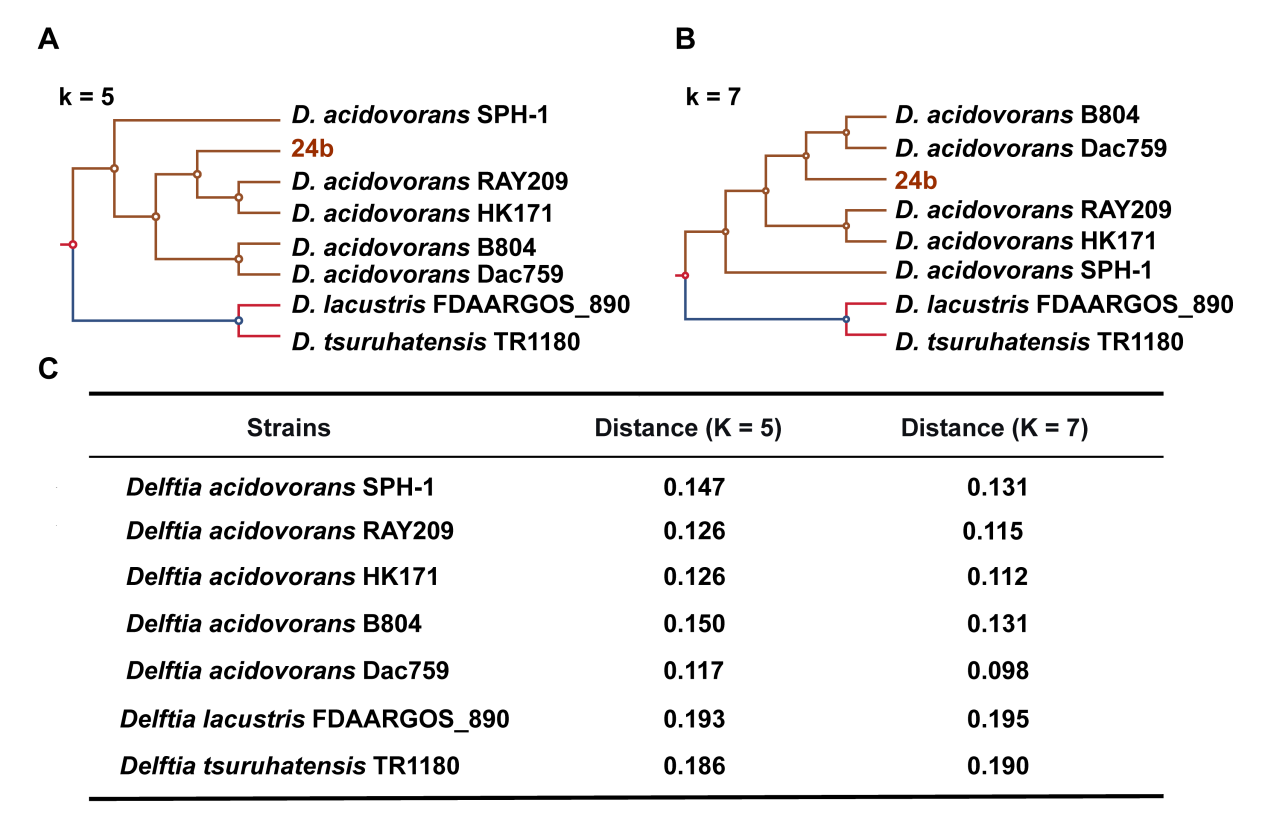
**

**Supplementary Figure S4 CVTree4-based phylogenetic trees and genetic distances of *Delftia* strains under k = 5 and k = 7.** (A) Phylogenetic tree at k = 5. (B) Phylogenetic tree at k = 7. (C) Genetic distances between strain 24b and other strains at k = 5 and k = 7. Outgroup species: *Delftia lacustris* FDAARGOS_890 and *Delftia tsuruhatensis* TR1180.
